# Predatory publishing lists: a systematic review

**DOI:** 10.1097/MS9.0000000000004733

**Published:** 2026-01-09

**Authors:** Fahmi H. Kakamad, Ayman M. Mustafa, Berun A. Abdalla, Shvan H. Mohammed, Sasan M. Ahmed, Hiwa O. Abdullah, Jaafar O. Ahmed, Fakher Abdullah, Sarhang S. Abdalla, Tomas M. Mikael, Hunar A. Hassan, Kayhan A. Najar, Diyar A. Omar

**Affiliations:** a Scientific Affairs Department, Smart Health Tower, Sulaymaniyah, Iraq; b Kscien Organization for Scientific Research (Middle East office), Sulaymaniyah, Iraq; c College of Medicine, University of Sulaimani, Sulaymaniyah, Iraq; d Psychology Department, Faculty of Arts, Soran University, Soran, Iraq; e Medical Laboratory Technology, Shaqlawa Technical College, Erbil Polytechnic University, Erbil, Iraq

**Keywords:** Beall list, Cabell list, Kscien list, predatory journal, predatory publisher

## Abstract

The rise of predatory journals threatens the integrity of academic publishing by exploiting open-access models and bypassing rigorous peer review. The lack of standardized criteria complicates their identification. This study systematically reviews existing predatory journal lists, assessing their effectiveness in enhancing transparency and safeguarding scholarly publishing. This systematic review adhered to PRISMA guidelines, including lists identifying predatory journals from peer-reviewed sources or reputable organizations. Using relevant keywords, a comprehensive search was conducted across academic databases (PubMed, Web of Science, Scopus, DOAJ), grey literature, and publisher websites. Key variables extracted included governance, accessibility, update mechanisms, and identification criteria. A comparative analysis assessed transparency, evaluation processes, and gaps such as historical tracking and evolving criteria. Descriptive statistics, including frequency, percentage, median, and range, were calculated using SPSS Version 26.0. Ten lists identifying predatory journals were analyzed; six (60.0%) were established after 2017, and nine (90.0%) were publicly accessible. The majority (seven, 70.0%) covered journals and publishers, with nine (90.0%) relying on a manual review process for identification. Delisting criteria were unclear in eight (80.0%) of the lists. Most lists (six, 60.0%) were available in database format . In terms of updating frequency, one list (10.0%) was updated daily, and six lists (60.0%) did not specify their update frequency. While these lists help identify fraudulent publishing practices, criteria, updates, and delisting inconsistencies reduce their reliability. Standardized methodologies, transparency, and sustained efforts are needed to keep them relevant, ensuring they safeguard academic integrity and guide researchers toward credible publishing.

## Introduction

In recent years, the proliferation of predatory journals has become a significant concern within the academic community, undermining the integrity of scholarly publishing. These journals exploit the “publish or perish” pressure in academia by offering publication opportunities without the rigorous standards typically expected in legitimate academic publishing. They often promise publication without proper peer review, editorial oversight, or quality control while demanding substantial fees in return^[1,2]^. This issue has been exacerbated by the rise of open-access publishing, which, although democratizing access to research, has also created opportunities for fraudulent entities to exploit the academic publishing model without adhering to its ethical standards^[3]^.

**HIGHLIGHTS**

- Predatory journals exploit academic pressure by offering publication without proper peer review or editorial standards, often for high fees.
- The rise of open-access publishing has inadvertently facilitated the growth of such journals.
- Beall’s List was removed in 2017; an anonymous scholar now maintains a similar resource.
- Several warning lists have been developed to combat the issue, but their effectiveness and comprehensiveness vary.
- The current study seeks to review and evaluate these lists to support efforts in addressing predatory publishing.

The term “predatory publishing” was first coined by Jeffrey Beall, who created a list of such journals, which became a crucial resource for researchers navigating the complex landscape of academic publishing. Beall’s List identified key characteristics of predatory journals, such as misleading claims about indexing and impact factors, lack of transparency in editorial practices, and fabricated editorial boards^[4]^. Beall’s List was removed in 2017, likely in response to legal challenges from publishers included in the list. Anonymous scholars maintains and updates the list on a separate platform^[5]^. Despite growing awareness of predatory publishing, no universally accepted criteria exist to define predatory journals. This lack of standardization complicates the identification process, leading to confusion and varying interpretations within the academic community.

The challenges of predatory journals extend beyond individual researchers to academic institutions and the broader scientific community. Their rapid proliferation threatens the integrity of scientific research and facilitates the spread of pseudoscience. Furthermore, the absence of standardized criteria further complicates the identification process, exacerbating confusion and inconsistency. Various efforts have been made to combat predatory publishing, including releasing several warning lists^[4,6,7]^. The current study aims to systematically review these lists by analyzing their strengths and weaknesses and assessing their effectiveness and comprehensiveness in identifying predatory journals. Through this analysis, the study seeks to provide insights into the utility of these lists and contribute to ongoing efforts to address predatory publishing in academia.

## Methods

### Study design and eligibility criteria

**Figure 1.**
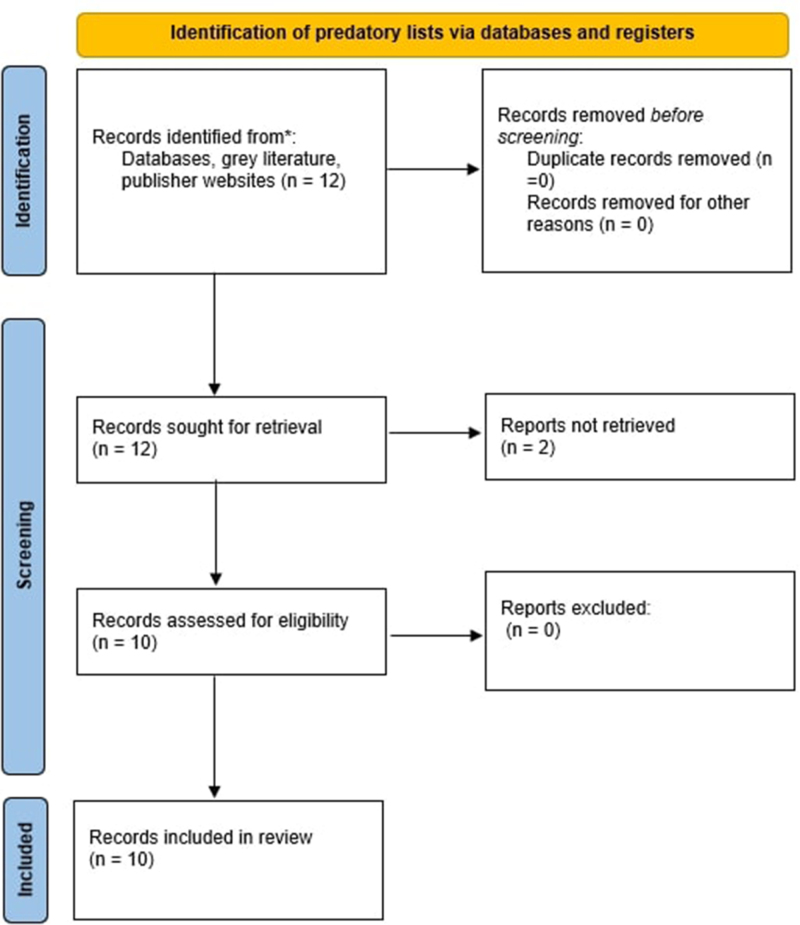
PRISMA flow diagram for list identification.

This systematic review followed the PRISMA guidelines to ensure transparency, rigor, and consistency (Fig. 1). This study also adhered to the Transparency in the Reporting of Artificial Intelligence (TITAN) 2025 guidelines^[8]^. The review included lists and databases explicitly designed to identify predatory journals and publishers, provided they were published in peer-reviewed sources or by reputable academic or nonprofit organizations. Opinion-based sources, nonscientific platforms, and any lists not specifically targeting predatory publishing practices were excluded. Additionally, lists lacking detailed criteria or governance transparency were not considered for inclusion.

### Information sources and search strategy

A broad search strategy was employed to identify relevant lists for the review. The following sources were systematically searched: academic databases, including PubMed, Web of Science, Scopus, and the Directory of Open Access Journals; grey literature, such as reports and institutional publications available through online repositories; and publisher websites, specifically those hosting blacklists or directories related to predatory publishing. The search was conducted using the keywords “predatory journals,” “predatory publishers,” “journal blacklist,” and “academic publishing fraud.” No time restrictions were imposed on the search, allowing for the inclusion of all relevant lists available at the time of the review.

### Data extraction

The following key variables were systematically extracted from each identified list: establishment year, responsible entity, management, accessibility, number of publishers and journals, updating date, primary source, content overview, number and type of criteria for predatory identification, stinging process, method of adding items, criteria for delisting, historical tracking, changes in criteria over time, community feedback and reporting, and data format. The extraction process aimed to capture relevant details regarding each list’s creation, maintenance, criteria, and accessibility. Detailed information was recorded for variables where applicable, including governance structures, update frequencies, and the mechanisms for adding or removing items.

### Data synthesis and analysis

A comparative analysis assessed how each list identifies predatory journals, manages transparency, and handles updates and removals. Key criteria, processes, and community feedback patterns were noted, along with gaps like historical tracking or evolving criteria. Descriptive statistics, including frequency, percentage, median, and range, were calculated using the Statistical Package for the Social Sciences (SPSS) Version 26.0.

## Results

Ten lists were identified, all dedicated to identifying predatory journals^[9–18]^. Most of these lists, six (60.0%), were established after 2017. Accessibility was predominantly public, with nine (90.0%) lists freely available to users. In terms of administrative framework, seven (63.6%) lists operated under a centralized model, while three (27.3%) adopted a decentralized approach, and one (9.1%) used a collaborative framework (Table 1). Regarding the scope of content, most lists seven (70.0%) identified predatory publishers and journals, while the remaining three (30.0%) focused exclusively on predatory journals. Only one list (10.0%) employed a stinging operation as part of its identification process, while the other nine (90.0%) relied on manual review and external reports to identify predatory entities (Table 2).

**Table 1:** General characteristics of predatory journal lists

List name	Temporal origin	Institutional oversight	Administrative framework	Accessibility	Last update	Updating frequency
Beall’s List^[9]^	2008	Independent scholar	Centralized	Public	Dec 2024	Not specified
Original version						
Current version						
		Anonymous	Decentralized			
Cabell’s List^[10]^	2017	Commercial entity	Centralized	Subscription-based	Dec 2024	Not specified
Predatory Reports^[11]^	Unknown	Unknown	Decentralized	Public	Mar 2024	Not specified
Kscien’s List^[12]^	2020	Research organization	Collaborative	Public	Ongoing	Daily
Kanalregister^[13]^	2010	Governmental body	Centralized	Public	Jan 2025	Annually
Open Access Journal list^[14]^	2017	Governmental body	Centralized	Public	Jan 2024	Not specified
Early warning list^[15]^	2020	Academic institution	Centralized	Public	Feb 2024	Annually
Journal insights predatory list^[16]^	2024	Commercial entity	Centralized	Public	Oct 2024	Not specified
International Journal Blacklist^[17]^	Unknown	Commercial entity	Centralized	Public	Not specified	Monthly
Predatory list^[18]^	2024	Not specified	Decentralized	Public	Nov 2024	Not specified

**Table 2:** Scope and content of predatory journal lists

List name	No. of publishers	No. of journals	Contents	No. of criteria	Stinging operation	Community involvement
Beall’s List			Predatory publishers, stand-alone journals, hijacked journals, misleading metrics	27	No	No
Original version	1163	1478				
Current version	1362	1529				
Cabell’s List	Over 16 000	Over 19 000	Predatory Reports, Journalytics	74	No	Yes
Predatory Reports	1361	2779	Predatory publishers, stand-alone journals	NA	No	Yes
Kscien’s List	1268	1700	Predatory publishers, stand-alone journals, hijacked journals, misleading metrics, predatory conferences	17	Yes	Yes
Kanalregister	1601	8655	Predatory publishers, predatory journals	NA	No	No
Open Access Journal list	Zero	1367	Predatory journals	NA	No	Yes
Early warning list	Zero	24	Predatory journals	NA	No	No
Journal insights predatory list	1324	1489	Predatory publishers, predatory journals	NA	No	No
International Journal Blacklist	Zero	29 020	Faulty journals	426	No	Yes
Predatory list	1333	1697	Predatory publishers, standalone journals, hijacked journals	NA	No	Yes

NA, not available.

Regarding adding items to the lists, nine (90.0%) used a manual review process, with one (10.0%) not specifying its inclusion methodology. The delisting criteria were poorly documented in eight (80.0%) lists, indicating a lack of transparency in the removal process. Only one list (10.0%) provided clearly defined delisting criteria. Beall’s List was cited as the primary source by four (40.0%) lists, while five (50.0%) lists did not specify a primary source, and one (10.0%) cited multiple sources (Table 3). In terms of updating frequency, one list (10.0%) was updated daily, two lists (20.0%) were updated annually, and six lists (60.0%) did not specify their updating frequency. Concerning the identification criteria, four (40.0%) lists had specified criteria for identifying predatory journals, whereas six (60.0%) did not provide specific criteria. For data format, six (60.0%) lists employed a database format, three (30.0%) used simple webpages, and one (10.0%) relied on a PDF file. The meadian number of publishers across the identified lists was 1362, ranging from 1324 to 16 000 (Table 4).

**Table 3:** Methodology and documentation practices

List name	Method of adding items	Delisting criteria	Adapting criteria	Appeal process	Data format	Primary source
Beall’s List	Manual review process	Not well documented	Yes	Yes	Database	Beall idea
Cabell’s List	Manual review process	Not well documented	Yes	Yes	Database	Beall’s List
Predatory Reports	Manual review process	Not well documented	NA	No	Simple webpage	Unknown
Kscien’s List	Manual review process	Not well documented	Yes	Yes	Database	Beall’s List
Kanalregister	NA	Not well documented	Yes	Yes	Database	Unknown
Open Access Journal list	Manual review process	Not well documented	NA	NA	PDF file	Unknown
Early warning list	Manual review process	Not well documented	No	Yes	Simple Webpage	Unknown
Journal insights predatory list	NA	Not well documented	NA	No	Simple webpage	Beall’s List
International Journal Blacklist	Manual review process	Yes	Yes	Yes	Database	Unknown
Predatory list	Manual review process	Not well documented	NA	No	Database	Beall’s List + stop predatory journals

NA, not available.

**Table 4:** Characteristics and statistical summary of predatory journal lists

Variables	Frequency (%)
Administrative framework^a^	
Centralized	7 (63.6)
Decentralized	3 (27.3)
Collaborative	1 (9.1)
Accessibility	
Public	9 (90.0)
Subscription-based	1 (10.0)
Updating frequency	
Daily	1 (10.0)
Annually	2 (20.0)
Monthly	1 (10.0)
Not specified	6 (60.0)
Criteria for identification	
Specified	4 (40.0)
Not specified	6 (60.0)
Stinging operation	
Yes	1 (10.0)
No	9 (90.0)
Data format	
Database	6 (60.0)
Simple webpage	3 (30.0)
PDF file	1 (10.0)
Delisting criteria	
Not well documented	9 (90.0)
Clearly specified	1 (10.0)
No. of publishers, median (min–max)	1362 (1324–16 000)
No. of journals, median (min–max)	1489 (24–29 020)

^a^ Beall’s List (current and previous versions) was considered separately in this analysis.

## Discussion

As widely acknowledged, one of the primary measures of academic success is publishing in reputable scientific outlets, such as journals and conferences^[19]^. Over time, the model for funding scientific publications has shifted mainly towards open-access publishing. Open-access journals provide readers with unrestricted access to research, allowing authors to share their work with a broader audience than journals with paywalls, which can lead to higher citation rates. However, some unscrupulous publishers use the open-access model by creating deceptive websites and luring inexperienced researchers into paying for rapid publication. These publishers often send unsolicited emails to solicit submissions and conduct minimal or even fraudulent peer review processes, all to maximize profits by accepting as many manuscripts as possible. As open-access publishing has grown in popularity, predatory publishers have increased significantly^[20]^.

Beall’s List, created in 2008 by American librarian Jeffrey Beall, was initially compiled after he received numerous unsolicited invitations to join editorial boards of questionable journals. Though it initially attracted little attention, the list gained widespread recognition within the academic community by mid-2010. Beall’s List categorized entries into four groups: suspicious publishers, predatory standalone journals, hijacked journals, and journals with falsified metrics^[21]^. Despite its widespread use, the list faced criticism for its subjective judgment, particularly in journals such as *Frontiers*, which lacked clear, objective criteria for listing. Beall took down the list in early 2017 without providing a specific reason, and it has since been updated sporadically by anonymous successors^[22]^. In the present systematic review of predatory lists, it was found that 60% were established after 2017, indicating both the increasing awareness of predatory publishing in recent years and the ongoing efforts of the scientific community to address these practices. Additionally, among the identified lists, Beall’s List was cited as the primary source in 40.0% of lists, underscoring its continued influence.

In his 2013 article “Who’s Afraid of Peer Review,” John Bohannon conducted a large-scale sting operation to expose predatory publishing. He submitted 304 fabricated scientific papers containing critical methodological errors to various open-access journals, listing fictitious authors from nonexistent African institutions. Despite the papers’ obvious flaws, 157 journals (51.6%) accepted them, 98 (32.2%) rejected them, and 49 (16.1%) remained inactive or under review. Among 255 processed manuscripts, 60% showed no evidence of peer review. Notably, India had the highest acceptance rate (64 acceptances, 15 rejections), followed by the US (29 acceptances, 26 rejections)^[23]^. These findings underscored sting operations’ effectiveness in revealing predatory journals’ shortcomings. However, in the present systematic review, only one list (Kscien’s List) incorporated a sting operation as part of its identification process. In contrast, the remaining nine (90.0%) relied solely on manual review and predefined criteria to identify predatory entities.

Kscien’s List, established by the Kscien Organization, a nonprofit founded by young researchers from Iraq’s Kurdistan region, aims to promote research culture in developing countries and combat predatory publishing. In response to the discontinuation of Beall’s List, Kscien launched its predatory list in 2020, managed by a committee of 25 researchers who continuously update it to monitor evolving predatory tactics. Initially based on Beall’s four categories – predatory publishers, standalone journals, hijacked journals, and misleading metrics – the list has since expanded^[20]^. As predatory journals adopt more sophisticated strategies, such as professional website design, securing indexing, fabricating archives, and strengthening plagiarism checks, Kscien has broadened its scope. To enhance transparency, it introduced the Conference List to identify predatory conferences and the Cumulative List to systematically document journals affiliated with predatory publishers, addressing previous limitations in journal identification^[22]^.

Identifying predatory journals and publishers remains a critical issue in academic publishing, with accessibility varying between freely available lists and subscription-based models. While most lists (90.0%) in the present systematic review were publicly accessible, Cabell’s Predatory Reports was the only subscription-based resource, potentially creating barriers for researchers with limited funding and exacerbating inequalities in access to reliable scholarly publishing information^[6]^. Following the discontinuation of Jeffrey Beall’s List in 2017, Cabell Publishing Co. emerged as an alternative, initially developing its lists with input from Beall and incorporating elements of his work^[7]^. Unlike Beall’s List, which relied on subjective criteria and external input of varying validity, Cabell’s adopted a structured approach, using 74 criteria as outlined in version 1.1^[10]^. The company initially maintained two lists – a safelist and a blacklist – curated by an undisclosed team. In mid-2020, these lists were rebranded as *Journalytics* and *Predatory Reports*^[24]^. However, accessibility remains a concern. Da Silva *et al* highlighted that individual researchers cannot directly subscribe to *Predatory Reports*, restricting access to those with institutional support or financial resources. Additionally, no information is available regarding discounts for institutions in low- and middle-income countries. The absence of updates on the company’s website suggests that its criteria have remained unchanged since 2019^[7]^.

The updating frequency of lists identifying predatory journals is critical in ensuring their reliability and relevance. In the current analysis, it was observed that only one list was updated daily. This inconsistency poses significant risks, as outdated information can mislead researchers and leave them vulnerable to exploitation. The rapid establishment of new predatory journals further exacerbates this issue^[25]^. Predatory publishers frequently emerge and adapt to exploit evolving publishing landscapes, making it essential for these lists to be updated regularly to reflect current realities. Studies have shown that many predatory journals publish a significantly higher number of articles than non-predatory ones, indicating a troubling trend where authors are increasingly targeted by these entities^[2]^. As the academic community continues to grapple with the issue of predatory publishing, it is imperative to prioritize regular updates and improve transparency in updating practices. Addressing resource limitations and working towards standardized protocols for maintaining these lists will protect researchers from exploitation and safeguard scholarly integrity in an increasingly complex publishing environment^[1]^.

One limitation of this study is the potential for selection bias in identifying and including predatory journal lists, as some lesser-known or region-specific lists may have been missed despite comprehensive search efforts. Additionally, the study relies on available documentation and transparency of each list, meaning that internal decision-making processes and unpublished criteria could not be fully assessed, which may affect the completeness of the analysis.

## Conclusion

While these lists are essential in identifying fraudulent publishing practices, inconsistencies in criteria, update frequency, and delisting mechanisms limit their reliability. The findings underscore the need for standardized methodologies, greater transparency, and sustained efforts to ensure these lists remain accurate and relevant. Strengthening these aspects will enhance their role in safeguarding academic integrity and guiding researchers toward credible publishing avenues.

## Data Availability

The data are available from the corresponding author upon reasonable request.

## References

[1] Richtig G Berger M Lange-Asschenfeldt B. Problems and challenges of predatory journals. J Eur Acad Dermatol Venereol 2018;32:1441–49. 29729106 10.1111/jdv.15039 PMC6174996

[2] Kakamad FH Abdalla BA Abdullah HO. Lists of predatory journals and publishers: a review for future refinement. Eur Sci Edit 2024;50:e118119.

[3] Shen C Shah L. Predatory publishing practices: what researchers should know before submitting their manuscript. Insights 2023;36. doi:10.1629/uksg.631.

[4] Beall J. “Predatory” open-access scholarly publishers. Charlest Advis 2010;11:10–17.

[5] Strinzel M Severin A Milzow K. Blacklists and whitelists to tackle predatory publishing: a cross-sectional comparison and thematic analysis. MBIO 2019;10:10–128. 10.1128/mBio.00411-19 PMC6550518 31164459

[6] Abdullah HO Abdalla BA Kakamad FH. Predatory publishing lists: a review on the ongoing battle against fraudulent actions. Barw Medl J 2024. doi:10.58742/bmj.v2i2.91.

[7] da Silva JA Moradzadeh M Yamada Y. Cabells’ Predatory Reports criteria: assessment and proposed revisions. J Acad Libr 2023;49:102659.

[8] Agha RA Mathew G Rashid R. Transparency in the reporting of artificial intelligence–the TITAN guideline. Prem J Sci 2025;10:100082.

[9] Beall J Beall’s list of potential predatory journals and publishers. 2021. Accessed 10 March 2025. Available at: https://beallslist.net/#

[10] Cabells. Predatory Reports Criteria v1.1. 2019. Accessed 10 March 2025. Available at: https://blog.cabells.com/2019/03/20/predatoryreport-criteria-v1-1/

[11] Predatory Reports. Predatory Reports, about Us. 2023. Accessed 10 March 2025. Available at: https://predatoryjournals.org/the-list

[12] Kscien Organization. Kscien Organization website. 2018. Predatory publishing; Available at: Accessed 10 March 2025. https://kscien.org/predatory.php

[13] Kanalregister. Norwegian Register for Scientific Journals, Series, and Publishers. 2024. Accessed 10 March 2025. Available at: https://kanalregister.hkdir.no/sok?option=journals&input=

[14] Open Access Journal. Predatory Journals List. 2017. Accessed 10 March 2025. Available at: https://www.openacessjournal.com/blog/predatory-journals-list/

[15] Fenqubiao Early Warning List. Early Warning Journal List. 2020. Accessed 10 March 2025. Available at: https://earlywarning.fenqubiao.com/#/en/

[16] Journals Insights. Predatory Journals List. 2024. Accessed 10 March 2025. Available at: https://journalsinsights.com/predatory-journals-list

[17] Journal Index. International Journals Blacklist. Accessed 10 March 2025. Available at: https://journal-index.org/index.php/international-journals-blacklist#gsc.tab=0

[18] Predatory List. Journals. 2024. Accessed 10 March 2025. Available at: https://predatorylist.com/journals/

[19] Asadi A. Invitation to speak at a conference: the tempting technique adopted by predatory conferences’ organizers. Sci Eng Ethics 2019;25:975–79. 29520691 10.1007/s11948-018-0038-0

[20] Kakamad FH Mohammed SH Najar KA. Kscien’s list; a new strategy to hoist predatory journals and publishers. Int J Surg Open 2019;17:5–7.

[21] Beall J. Ban predators from the scientific record. Nature 2016;534:326. 10.1038/534326a 27306178

[22] Muhialdeen AS Ahmed JO Baba HO. Kscien’s list; a new strategy to discourage predatory journals and publishers. Barw Med 2023;1:1–3.

[23] Bohannon J. Who’s afraid of peer review? Science 2013;342:60–65. 24092725 10.1126/science.2013.342.6154.342_60

[24] Cabells. Announcement regarding brand-wide language changes, effective immediately. 2020. Accessed 10 March 2025. Available at: https://blog.cabells.com/2020/06/08/announcement/

[25] Bartholomew RE. Science for sale: the rise of predatory journals. J R Soc Med 2014;107:384–85. 25271271 10.1177/0141076814548526 PMC4206639

